# Genome Sequence of the Clinical Isolate Staphylococcus aureus subsp. aureus Strain UAMS-1

**DOI:** 10.1128/genomeA.01584-14

**Published:** 2015-02-12

**Authors:** Mohamed Sassi, Deepak Sharma, Shaun R. Brinsmade, Brice Felden, Yoann Augagneur

**Affiliations:** a University of Rennes 1, Inserm U835 Biochimie Pharmaceutique, Rennes, France; b Georgetown University, Department of Biology, Washington, DC, USA

## Abstract

We report here the draft genome sequence of Staphylococcus aureus subsp. aureus strain UAMS-1. UAMS-1 is a virulent oxacillin-susceptible clinical isolate. Its genome is composed of 2,763,963 bp and will be useful for further gene expression analysis using RNA sequencing (RNA-seq) technology.

## GENOME ANNOUNCEMENT

Staphylococcus aureus is an opportunistic human bacterial pathogen responsible for nosocomial and community-associated infections. S. aureus subsp. aureus strain UAMS-1 was originally isolated from the bone of a patient suffering from osteomyelitis (1), and although it is a methicillin-susceptible S. aureus (MSSA) and oxacillin-susceptible S. aureus (OSSA) strain (2), UAMS-1 is generally considered to be most closely related to methicillin-resistant S. aureus strain MRSA252, whose genome was completed in 2004 (3, 4). While this strain is widely used by the community along with S. aureus strains RN1, Newman, COL, and USA300 (5), there are currently no genomic data available for UAMS-1. Here, we report the genome sequencing of strain UAMS-1, which is a prerequisite for sophisticated physiological or RNA-seq-based gene expression studies.

Genomic DNA was isolated from strain UAMS-1 grown in tryptic soy broth medium (5 ml) at 37°C using the Wizard genomic DNA purification kit (Promega), according to the manufacturer’s recommendations for efficient lysis of S. aureus, washing it extensively with isopropanol and ethanol. Subsequently, the genomic DNA was precipitated with sodium acetate and washed twice with ethanol (70% [vol/vol]). DNA was sheared to yield ~600-bp fragments using an M220 focused ultrasonicator (Covaris). Unwanted small and large fragments were removed by size selection using AMPure XP beads (Beckman Coulter). The DNA library was prepared using the NEBNext Ultra DNA library prep kit for Illumina (NEB) and sequenced as paired-end reads using an Illumina MiSeq platform and the MiSeq reagent kit version 3 (600 cycles; Illumina, Inc.). The run generated 2,178,114 reads (630 Mb of sequence data) with 110× coverage.

The Illumina reads were trimmed using Trimmomatic (6), quality filtered using the Fastx-toolkit (http://hannonlab.cshl.edu/fastx_toolkit/), and then assembled using the SPAdes software (7, 8). GapFiller version 1.10 (9, 10) was used to improve the initial set of contigs, and the genome sequence of strain MRSA252 was used as the reference to order and orient the contigs. The draft genome sequence of S. aureus UAMS-1 yielded 2 scaffolds of 7 contigs containing 2,763,963 bp. The G+C content is 32.71%.

The genome of UAMS-1 was annotated using the NCBI Prokaryotic Genome Annotation Pipeline (PGAP) (11). The genome contains 2,808 genes, including 9 rRNAs (5S, 16S, and 23S), 60 tRNAs, and 86 pseudogenes. A total of 2,653 genes (94.5%) encode putative proteins that represent a coding capacity of 2,611,945 bp. Among these genes, 515 (19.41%) are annotated as encoding hypothetical proteins. Using the PHAge Search Tool (PHAST) (12) and CRISPRFinder (13), we detected one intact and complete Staphylococcus phage and five possible clustered regularly interspaced short palindromic repeats (CRISPRs), respectively. Finally, the average nucleotide identity between UAMS-1 and MRSA252 is 97.62%, suggesting that although they are considered to be closely related by the scientific community, there are potentially substantial differences between these two strains.

### Nucleotide sequence accession numbers.

The S. aureus subsp. aureus UAMS-1 genome shotgun project has been deposited at DDBJ/EMBL/GenBank under the accession no. JTJK00000000. The version described in this paper is version JTJK01000000.
